# Hydrogen Peroxide Induces α-Tubulin Detyrosination and Acetylation and Impacts Breast Cancer Metastatic Phenotypes

**DOI:** 10.3390/cells12091266

**Published:** 2023-04-27

**Authors:** Megan B. Stemberger, Julia A. Ju, Keyata N. Thompson, Trevor J. Mathias, Alexandra E. Jerrett, Katarina T. Chang, Eleanor C. Ory, David A. Annis, Makenzy L. Mull, Darin E. Gilchrist, Michele I. Vitolo, Stuart S. Martin

**Affiliations:** 1 Graduate Program in Biochemistry and Molecular Biology, University of Maryland School of Medicine, 108 N. Greene St., Baltimore, MD 21201, USA; 2 Graduate Program in Molecular Medicine, University of Maryland School of Medicine, 800 W. Baltimore St., Baltimore, MD 21201, USA; 3 Marlene and Stewart Greenebaum NCI Comprehensive Cancer Center, University of Maryland School of Medicine, 22 S. Greene St., Baltimore, MD 21201, USA; 4 Graduate Program in Epidemiology and Human Genetics, University of Maryland School of Medicine, 655 W. Baltimore St., Baltimore, MD 21201, USA; 5 Departments of Pharmacology and Physiology, University of Maryland School of Medicine, 655 W. Baltimore St., Baltimore, MD 21201, USA; 6 United States Department of Veterans Affairs, VA Maryland Health Care System, 10 18 N. Greene St., Baltimore, MD 21201, USA

**Keywords:** tumor microenvironment, reactive oxygen species, microtubules, microtentacles

## Abstract

Levels of hydrogen peroxide are highly elevated in the breast tumor microenvironment compared to normal tissue. Production of hydrogen peroxide is implicated in the mechanism of action of many anticancer therapies. Several lines of evidence suggest hydrogen peroxide mediates breast carcinogenesis and metastasis, though the molecular mechanism remains poorly understood. This study elucidates the effects of exposure to elevated hydrogen peroxide on non-tumorigenic MCF10A mammary epithelial cells, tumorigenic MCF7 cells, and metastatic MDA-MB-231 breast cancer cells. Hydrogen peroxide treatment resulted in a dose- and time-dependent induction of two α-tubulin post-translational modifications—de-tyrosination and acetylation—both of which are markers of poor patient prognosis in breast cancer. Hydrogen peroxide induced the formation of tubulin-based microtentacles in MCF10A and MDA-MB-231 cells, which were enriched in detyrosinated and acetylated α-tubulin. However, the hydrogen peroxide-induced microtentacles did not functionally promote metastatic phenotypes of cellular reattachment and homotypic cell clustering. These data establish for the first time that microtentacle formation can be separated from the functions to promote reattachment and clustering, which indicates that there are functional steps that remain to be identified. Moreover, signals in the primary tumor microenvironment may modulate α-tubulin post-translational modifications and induce microtentacles; however, the functional consequences appear to be context-dependent.

## 1. Introduction

Significant progress has been made in early detection and treatment of breast cancer; however, metastasis remains the leading cause of patient mortality [1]. Technical challenges associated with studying the metastatic cascade have limited our ability to identify the targetable cellular changes underlying this fatal process [2]. As a result, most current anticancer therapies target the growth of primary tumors. While effective at slowing primary tumor growth, these treatments often result in a broad side effect profile [3] and provide limited benefit for patients with metastatic disease [1]. There is an urgent need for new research to uncover changes that occur both within cancer cells and the surrounding tumor microenvironment (TME) that drive metastasis and could ultimately lead to better therapeutic targets.

In addition to cell intrinsic changes, alterations in the local TME, such as elevated levels of reactive oxygen species (ROS), synergize with cancer cells to drive oncogenesis [4]. Hydrogen peroxide (H_2_O_2_) is one of the major ROS that has well-characterized roles in physiological processes, such as growth and metabolism [5], and is aberrantly produced in pathologies including neurodegenerative diseases, chronic inflammation, and cancer [4,6]. The normal intracellular concentration of H_2_O_2_ is between 1–100 nM [5], while levels in the TME are much higher, with one report stating that levels may reach 50–100 µM [7]. The biological effects of H_2_O_2_ are context-dependent and can be pro- or anti-tumorigenic depending on factors including cell type and concentration [4,8]. In breast cancer, H_2_O_2_ has been reported to promote changes in tumor cells, such as activating the epithelial–mesenchymal transition (EMT), causing genomic instability, and driving apoptotic resistance [4]. H_2_O_2_ also primes the TME by altering cancer-associated fibroblasts, tumor-associated endothelial cells, and T cells [4]. However, excessive H_2_O_2_ exposure can trigger irreversible cytotoxicity; thus, cancer cells maintain tight ROS regulation by upregulating antioxidant pathway genes [8,9]. As a result, oxidative stress is sufficiently elevated to promote tumorigenesis and cancer progression without becoming high enough to cause cytotoxicity.

ROS are also therapeutically relevant as many anticancer therapies exert their effect through a mechanism involving ROS generation [9,10,11,12]. A common target of existing breast cancer therapeutics is the microtubule network. These compounds include microtubule stabilizers, such as taxanes and epothilones, and microtubule destabilizers, such as vinca alkaloids [13]. Both classes of microtubule-targeting agents have been shown to increase the production of ROS [14]. However, broad microtubule stabilization, such as with taxanes, has been found to inadvertently increase the metastatic potential of breast tumor cells in vitro [15] and in vivo [16,17]. Rather than indiscriminately targeting all microtubules, a better therapeutic strategy may be to target specific subsets of modified microtubules that are unique to cancer cells. This strategy would potentially produce fewer side effects than current therapies, which indiscriminately target all microtubules in both healthy and cancer cells.

Microtubule heterogeneity in cells is achieved in part through post-translational modifications (PTMs) [18]. Two α-tubulin PTMs—detyrosination and acetylation—are of particular interest in breast cancer biology as both are elevated in increasingly aggressive breast cancer cell lines and serve as indicators of poor patient prognosis [19,20,21]. Detyrosination is the cleavage of the C-terminal tyrosine residue, while acetylation adds an acetyl group to lysine 40 [18]. Detyrosinated-tubulin (DeTyr-tub) promotes cell invasion in vitro [20], localizes at the invasive front of breast cancer patient tumor samples [22], and is correlated with poor patient outcomes [21]. Similarly, acetylated-tubulin (Acetyl-tub) promotes reattachment and migration of breast cancer cells in vitro [19]. Expression of Acetyl-tub is associated with the aggressive basal-like breast cancer subtype, with select patients that express higher acetylated-tubulin having significantly poorer survival rates [19]. Furthermore, when breast tumor cells are placed in a free-floating microenvironment analogous to what they would encounter during metastasis in vivo, these cells form microtubule-based protrusions termed microtentacles (McTNs), which are enriched in DeTyr-tub and Acetyl-tub [19,23]. Functionally, McTNs promote endothelial engagement (reattachment) [22] and homotypic cell clustering [24] in vitro. This fact is of high clinical relevance because circulating tumor cells (CTCs) that form clusters have up to 50x-higher metastatic efficiency in vivo compared to single CTCs [25]. Additionally, three independent genetic models of breast cancer metastasis have demonstrated that cells with increased McTNs have enhanced lung retention in vivo [26,27,28,29]. Moreover, disruption of McTNs using the FDA-approved microtubule destabilizer—Vinorelbine—reduces the metastatic efficiency of circulating breast tumor cells [24]. Altogether, this evidence highlights the importance of identifying factors that regulate McTN formation and function. 

To investigate the connection between ROS, microtubule PTMs, and breast cancer phenotypes, we treated three independent breast-derived cell lines—non-tumorigenic MCF10A, tumorigenic MCF7, and metastatic MDA-MB-231s—with H_2_O_2_. We observed a dose- and time-dependent induction of DeTyr-tub and Acetyl-tub, with no change in overall α-tubulin or cell viability within the experimental timeframe of our phenotypic assays. To define the functional significance of these H_2_O_2_ effects, we tested metastatic phenotypes of McTN formation, reattachment, and homotypic cell clustering. 

## 2. Materials and Methods

### 2.1. Cell Culture and Treatment

All cell lines were originally obtained from the American Type Culture Collection (ATCC, Manassas, VA, USA). MCF10A cells were maintained in DMEM/F12 (Invitrogen, Waltham, MA, USA, Cat. #10565-018) supplemented with 5% Horse Serum (Invitrogen, Waltham, MA, USA, Cat. #26050-088), 1% Penicillin/Streptomycin (Gemini Bio-Products, West Sacramento, CA, USA, Cat. #400-109), 20 ng/mL Human EGF (Gibco, Grand Island, NY, USA, Cat. #PHG0313), 10 µg/mL Insulin (Gemini Bio-Products, West Sacramento, CA, USA, Cat. #800-112), 0.5 µg/mL Hydrocortisone (Sigma-Aldrich, Burlington, MA, USA, CAS #H0135), and 0.1 µg/mL Cholera Toxin (Sigma-Aldrich, Burlington, MA, USA, CAS #9012-63-9). MCF7 and MDA-MB-231 cells were maintained in DMEM (Corning, Corning, NY, USA, Cat. #10-017-CV) supplemented with 10% Fetal Bovine Serum (Gemini Bio-Products, West Sacramento, CA, USA, Cat. #100-106) and 1% Penicillin/Streptomycin (Gemini Bio-Products, West Sacramento, CA, USA, Cat. #400-109).

### 2.2. Reagents

Treatments were performed in Hanks’ Balanced Salt Solution (HBSS) with calcium (Thermo Fisher, Waltham, MA, USA, Cat. #14025092). Hydrogen peroxide (H_2_O_2_, 30% *w*/*w*) (Sigma-Aldrich, Burlington, MA, USA, CAS #H1009) was diluted in HBSS to indicated concentrations and solutions were applied directly to adherent cells and incubated for the indicated time periods at 37 °C. Wheat Germ Agglutinin 488 (WGA; Invitrogen, Waltham, MA, USA, Cat. #W11261) was used at a dilution of 1:100 in PBS to stain cell membranes and McTNs. NucBlue (Thermo Fisher, Waltham, MA, USA, Cat. #R37605) nuclear stain was used for live cell visualization and Hoechst 33342 (1:500–1:1000, Thermo Fisher, Waltham, MA, USA, Cat. #62249) was used to visualize nuclei in fixed cells. All antibodies, catalog numbers, and dilutions can be found in Section 2.3 and Section 2.4.

### 2.3. Immunoblotting

Cells were seeded at an initial density of 200,000–300,000 cells/well in 6-well tissue culture-treated dishes 48 h prior to experimentation. Following indicated treatments, adherent cells were washed with 1 X PBS, harvested in 1 X RIPA lysis buffer (Millipore, Burlington, MA, USA, Cat. #20-188) supplemented with protease inhibitor (Sigma-Aldrich, Burlington, MA, USA, CAS #S8820) and phosphatase inhibitor cocktail (Millipore, Burlington, MA, USA, Cat. #524625), and subjected to rocking at 4 °C and/or freeze-thaw to facilitate cell lysis. Whole cell lysates were centrifugated at 14,000× RPM for 20 min and supernatants were collected. Total protein concentrations were quantitated using the colorimetric Bio-Rad *DC* Protein Assay (Bio-Rad, Philadelphia, PA, USA, Cat. #5000113, 5000114, 5000115) and samples were diluted to equal protein concentrations. 

Equal protein mass and volume from each sample was loaded into 4–12% Bis-Tris Gels (Invitrogen, Waltham, MA, USA, Cat. #NP0335BOX) and separated using electrophoresis. All antibodies were diluted in 2.5% or 5% Bovine Serum Albumin (BSA) in 1 X tris-buffered saline supplemented with 0.5% TWEEN-20. Primary antibodies were anti-α-tubulin (1:1000, Cell Signaling Technologies, Danvers, MA, USA, Cat. #2144S), anti-acetylated-α-tubulin (1:1000, Cell Signaling Technologies, Danvers, MA, USA, Cat. #5335S), anti-detyrosinated-α-tubulin (1:10,000, ReVmAb Biosciences, South San Francisco, CA, USA, Cat. #31-1335-00), and anti-GAPDH (1:5000, Santa Cruz Biotechnology, Dallas, TX, USA, Cat. #sc-32233). HRP-conjugated secondary antibodies were used (1:5000, Jackson Labs, Bar Harbor, ME, USA, Cat. #711-035-152, 715-035-150) and proteins were visualized by incubating membranes with ECL (Thermo Fisher, Waltham, MA, USA, Cat. #32106) and imaging using the iBright system (Invitrogen, Waltham, MA, USA). Densitometry was performed using ImageJ and graphed in GraphPad Prism 9 (San Diego, CA, USA). 

### 2.4. Immunofluorescence

For attached immunofluorescence, cells were seeded at an initial density of 20,000–30,000 cells/well in 8-well Ibidi slides (Ibidi, Gräfelfing, Germany, Cat. #80826) 48 h prior to experimentation. For suspended immunofluorescence, cells were treated in adherent conditions, followed by tethering in TetherChips. Following indicated treatments, cells were fixed with 3.7% formaldehyde, permeabilized using 0.1% Triton-X (USB, Cleveland, OH, USA, CAS#9002-93-1) in 1 X PBS, and blocked for at least one hour in 5% BSA supplemented with 0.5% NP-40 substitute (Amresco, Solon, OH, USA, M158) in 1 X PBS. The following primary antibodies were diluted in 2.5% BSA supplemented with 0.5% NP-40 substitute in 1 X PBS and incubated overnight at 4 °C: anti-α-tubulin (Sigma-Aldrich, Burlington, MA, USA, CAS #T6199), anti-acetylated-α-tubulin (1:1000, Cell Signaling Technologies, Danvers, MA, USA, Cat. #5335S) and anti-detyrosinated-α-tubulin (1:10,000, ReVmAb Biosciences, South San Francisco, CA, USA, Cat. #31-1335-00). To visualize target proteins, cells were incubated for at least 1 h at room temperature with fluorophore-conjugated secondary antibodies (1:5000; Invitrogen, Waltham, MA, USA, Cat. #A-11001, A-11012) in 1 X PBS solution with Hoechst 33342 added (1:500–1:1000, Thermo Fisher, Waltham, MA, USA, Cat. #62249) to visualize nuclei. Slides were mounted using Fluoromount-G (Invitrogen, Waltham, MA, USA, Cat. #00-4958-02), imaged using an Olympus IX81 microscope with a Fluoview FV1000 confocal laser scanning system at 60× magnification or Nikon AX R confocal microscope, and visualized in ImageJ.

### 2.5. Trypan Blue Viability Assay

Cells were seeded at an initial density of 150,000 cells/well in 6-well tissue culture-treated dishes 24 h prior to experimentation. Cells were treated for 60 min with HBSS or 250 µM H_2_O_2_, after which treatment was removed and replaced with normal growth medium. At all indicated time points before and after treatment, the percentage viability of floating and adherent cells was determined through centrifuging the cell suspension, aspirating the supernatant, resuspending the pellet in growth medium, combining the cell suspension 1:1 with 0.4% Trypan Blue (Invitrogen, Waltham, MA, USA, Cat. #T10282), and recording live cell number and percentage cell viability using the Countess automated cell counter (Invitrogen, Waltham, MA, USA, Cat. #C10227). Each condition and time point was read at least twice and represented as the average value. 

### 2.6. Microtentacle Analysis 

Cells were seeded at an initial density of 200,000–300,000 cells/well in 6-well tissue culture-treated dishes 24 h prior to experimentation. Cells were treated for 60 min with HBSS or 250 µM H_2_O_2_, detached using TrypLE express (Gibco, Grand Island, NY, USA, Cat. #12604013), and seeded in technical triplicate on TetherChips as previously described [30], with the exception that the DOTAP lipid (Avanti Polar Lipids, Alabaster, AL, USA, Cat. #890890C) was added prior to thermal crosslinking to improve cell capture and tethering efficiency. Tethered cells were fixed using 3.7% formaldehyde in 1 X PBS, followed by membrane staining with Alexa Fluor™ 488 Conjugated Wheat Germ Agglutinin (WGA; 1:100, Invitrogen, Waltham, MA, USA, Cat. #W11261) and nuclear staining using Hoechst 33342 Solution (1:500–1000, Thermo Fisher, Waltham, MA, USA, Cat. #62249).

For assessment of McTN positivity, at least 100 random cells per channel were blindly scored as positive or negative for McTNs via visualization on a Nikon Ti2E fluorescence microscope. Positive cells were defined as having at least one McTN that is greater than the cell radius. Three technical replicates per condition, per each of three independent experiments, were scored and averaged. Data were presented as the mean ± SD of three independent experiments. Representative images were acquired on an Olympus IX81 microscope with a Fluoview FV1000 confocal laser scanning system at 60× magnification.

### 2.7. Cell Clustering Assay

Cells were seeded at an initial density of 200,000–300,000 cells/well in 6-well tissue culture-treated dishes 48 h prior to experimentation. Adherent cells were treated for 60 min with HBSS or 250 µM H_2_O_2_, followed by treatment washout, cell detachment, counting, and dilution to a final concentration of 800,000 cells/mL. For the next step, 40,000 cells/well were seeded in technical triplicates in two low-attach 96-well plates: one for T = 0 and the other for T = 6 h. For the T = 0 plate, one drop of NucBlue (Thermo Fisher, Waltham, MA, USA, Cat. #R37605) nuclear stain was added to each well and the entire contents of each well was added to one channel of a polyelectrolyte multilayer coated Ibidi microfluidic slide (Ibidi, Gräfelfing, Germany, Cat. #80601) and scanned on a Nikon Ti2E microscope at 4× magnification. For consistency, imaging was performed at equal exposure between control and treated conditions, as well as time points, for each independent experiment. The T = 6 h plate was left undisturbed for 6 h to allow for cell cluster formation, followed by the same nuclear staining and imaging protocol. Analysis of cluster number and area was performed in ImageJ. The same size region of each image was analyzed, images were manually thresholded to account for variability in staining intensity, and identical parameters for particle size were utilized (75–200,000 µm) to discern both single cells and cell clusters. Representative images were visualized in ImageJ.

### 2.8. Reattachment Assay

Cellular reattachment was assessed in real time using the xCELLigence RTCA instrument (Agilent, Santa Clara, CA, USA). Cells were seeded at an initial density of 200,000–300,000 cells/well in 6-well tissue culture-treated dishes 48 h prior to experimentation. Cells were treated for 60 min with HBSS or 250 µM H_2_O_2_, after which treatment was aspirated and cells were detached, counted, and resuspended in normal growth medium. For the next step, 40,000 cells/well were seeded in normal growth medium in at least technical triplicate in E-plates (Agilent, Santa Clara, CA, USA, Ref. #5469830001) and impedance measurements were taken every 5 min for 24 h. Data were presented as the mean ± SD of one representative experiment out of three independent experiments.

Time-lapse microscopy images were also taken of cells following the same treatment conditions to monitor reattachment to standard tissue culture-treated dishes. Cells were resuspended in standard growth medium and seeded in 24-well tissue culture-treated dishes at a density of 50,000 cells/well and phase-contrast images were taken every hour using a Nikon Ti2E microscope with a stage top Tokai-hit incubator at 10× magnification.

### 2.9. Statistical Analysis

Statistical analyses were performed in GraphPad Prism Version 9.4.1 (San Diego, CA, USA) and a *p*-value ≤ 0.05 was considered statistically significant. All Student’s *t*-tests were unpaired and two-tailed. One- and Two-Way ANOVA were performed with post-tests, as indicated in figure legends. 

## 3. Results

### 3.1. Hydrogen Peroxide Induces Rapid Post-Translational Modifications to Microtubules

**Figure 1:**
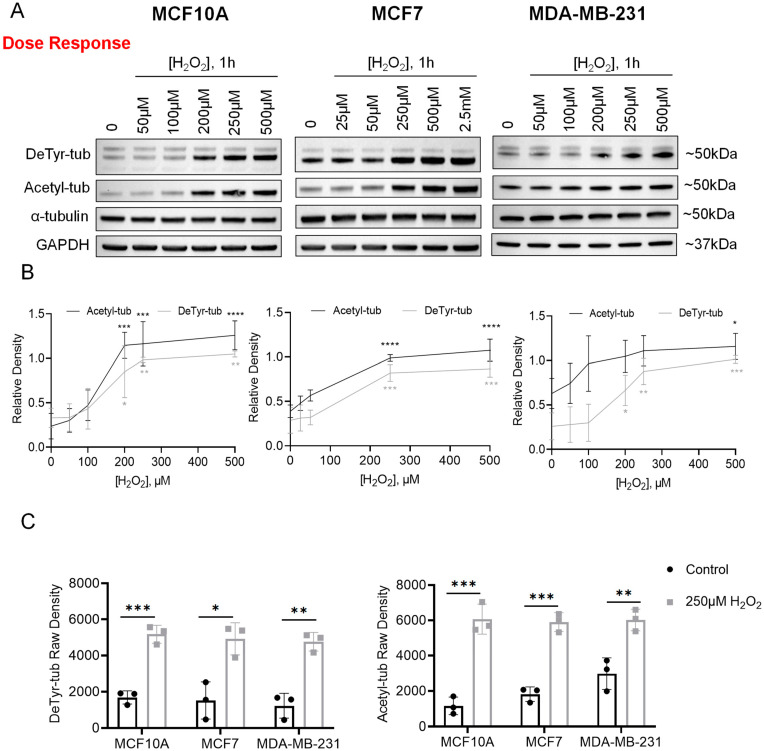
Hydrogen peroxide induces dose-dependent α-tubulin detyrosination and acetylation in non-tumorigenic, tumorigenic, and metastatic breast cancer cell lines. (**A**) Representative immunoblots following one hour treatment with control media (HBSS) or increasing doses of H_2_O_2_ in MCF10A cells (left), MCF7 cells (middle), and MDA-MB-231 cells (right), n = 3. (**B**) Densitometry analysis of three independent H_2_O_2_ dose response experiments (corresponding to Panel (**A**)) was performed in ImageJ following normalization of indicated proteins to total α-tubulin. Data are shown as mean ± SD, n = 3. Data were compared using a one-way ANOVA with Dunnett’s multiple comparisons test: no symbol indicates non-significant; *—*p* ≤ 0.05; **—*p* ≤ 0.01; ***—*p* ≤ 0.001; and ****—*p* ≤ 0.0001. (**C**) Raw density analysis of indicated proteins (left: DeTyr-tub; right: Acetyl-tub) from three independent H_2_O_2_ dose response experiments (corresponding to Panel (**A**)) was performed in ImageJ. Data are shown as mean ± SD, n = 3. For each independent cell line, control vs. treated populations were compared using an unpaired *t*-test: *—*p* ≤ 0.05; **—*p* ≤ 0.01; and ***—*p* ≤ 0.001.

To test the effects of ROS on the microtubule network, we treated three cell lines—MCF10A, MCF7, and MDA-MB-231—with doses of H_2_O_2_ similar to those found in the tumor microenvironment [7]. One hour treatment with concentrations of H_2_O_2_ between 50 µM and 500 µM revealed a dose-dependent increase in DeTyr-tub and Acetyl-tub (Figure 1A). Densitometry quantification was performed following normalization of DeTyr-tub and Acetyl-tub to total α-tubulin. In all three cell lines, one hour treatment with 250 µM H_2_O_2_ resulted in a statistically significant increase in the DeTyr-tub protein level (Figure 1B). Quantification of Acetyl-tub protein expression showed a similar trend, with significance reached in MCF10A and MCF7 cells; thus, 250 µM H_2_O_2_ was selected as the dose for future assays (Figure 1B). Raw densitometry values were plotted to directly compare DeTyr-tub and Acetyl-tub protein levels between control and 250 µM H_2_O_2_-treated cells (Figure 1C). A statistically significant induction of both DeTyr-tub and Acetyl-tub was observed in MCF10A, MCF7, and MDA-MB-231 cells (Figure 1C); thus, we selected 250 µM H_2_O_2_ as our dose for future experiments.

**Figure 2:**
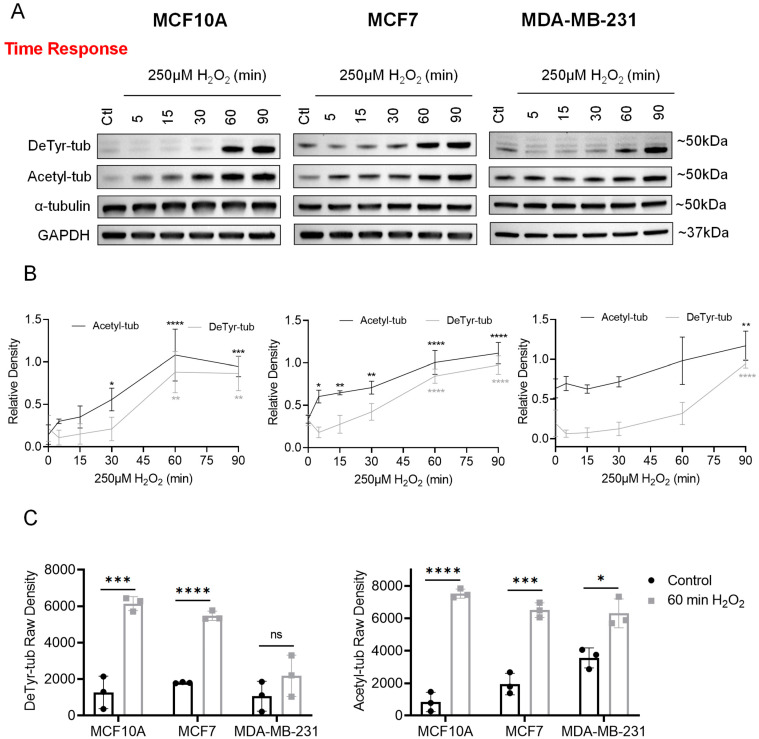
Hydrogen peroxide induces time-dependent α-tubulin detyrosination and acetylation in non-tumorigenic, tumorigenic, and metastatic breast cancer cell lines. (**A**) Representative immunoblots following a time course of 250 μM H_2_O_2_ treatment, n = 3. (**B**) Densitometry analysis of three independent H_2_O_2_ time course experiments (corresponding to Panel (**A**)) was performed in ImageJ following normalization of indicated proteins to total α-tubulin. Data are shown as mean ± SD, n = 3. Data were compared using a one-way ANOVA with Dunnett’s multiple comparisons test: no symbol indicates non-significant; *—*p* ≤ 0.05; **—*p* ≤ 0.01; ***—*p* ≤ 0.001; and ****—*p* ≤ 0.0001. (**C**) Raw density analysis of indicated proteins (left: DeTyr-tub; right: Acetyl-tub) from three independent H_2_O_2_ time course experiments (corresponding to Panel (**A**)) was performed in ImageJ. Data are shown as mean ± SD, n = 3. For each independent cell line, control vs. treated populations were compared using an unpaired *t*-test: ns—*p* > 0.05; *—*p* ≤ 0.05; ***—*p* ≤ 0.001; and ****—*p* ≤ 0.0001.

**Figure 3:**
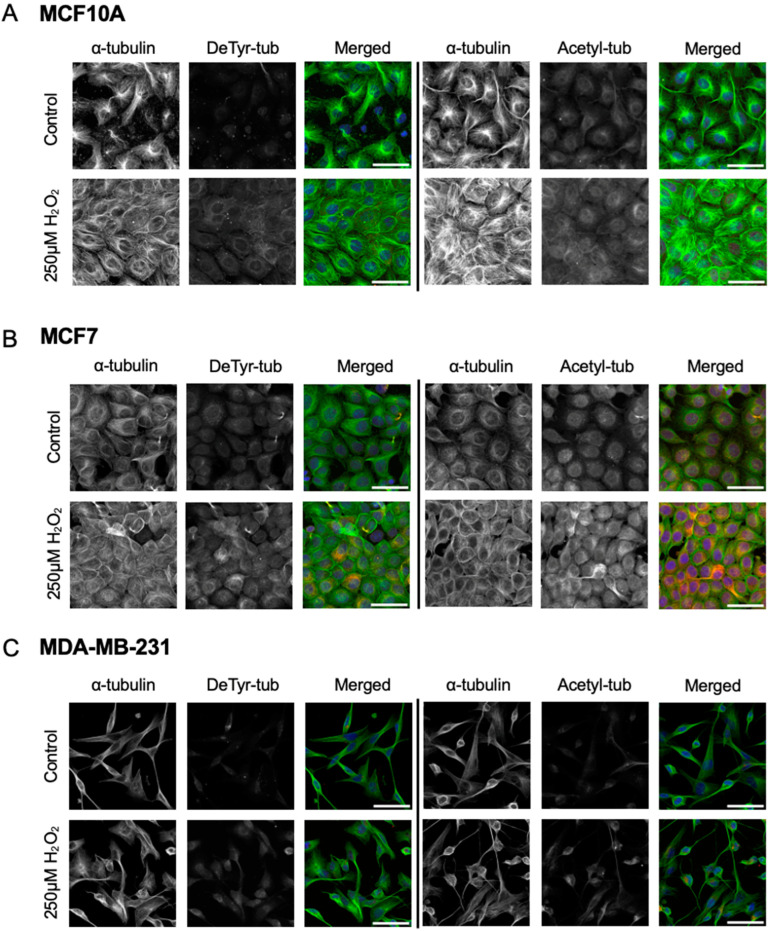
Hydrogen peroxide induces α-tubulin de-tyrosination and acetylation in non-tumorigenic, tumorigenic, and metastatic breast cancer cell lines. Immunofluorescence following one hour treatment of MCF10A (**A**), MCF7 (**B**), and MDA-MB-231 (**C**) cells with control (top row) or 250 μM H_2_O_2_. Slides imaged using a Nikon AX R confocal microscope (20×). Scale bar = 50 μm.

To ensure the experimental timeframe was optimal, a time course was performed to determine the effects of 250 µM H_2_O_2_ treatment at indicated time points between 5 and 90 min. Immunoblot results validated that 60 min was a reasonable timepoint to reliably observe both H_2_O_2_-induced tubulin PTMs (Figure 2A). Densitometry analysis was performed following normalization of DeTyr-tub and Acetyl-tub to total α-tubulin, confirming a statistically significant induction in DeTyr-tub and Acetyl-tub at 60 min in both MCF10A and MCF7 cell lines (Figure 2B). The MDA-MB-231 cells did not reach statistical significance until 90 min of treatment, likely owing to variance between replicates (Figure 2B). Given that Acetyl-tub induction reached statistical significance by 30 min in both MCF10A and MCF7 cells (Figure 2B), we decided to proceed with 60 min. Raw densitometry values were plotted and revealed a statistically significant increase in DeTyr-tub at 60 min in MCF10A and MCF7 cells, as well as a statistically significant increase in Acetyl-tub in all three cell lines following 60 min (Figure 2C). Finally, we examined the effects of H_2_O_2_ on the microtubule network using immunofluorescence (Figure 3). In line with the immunoblot findings, we observed an enrichment in the proportion of the α-tubulin network that was composed of DeTyr-tub and Acetyl-tub filaments in H_2_O_2_-treated MCF10A (Figure 3A), MCF7 (Figure 3B), and MDA-MB-231 (Figure 3C) cells compared to control cells. Overall, these data demonstrate that H_2_O_2_ induces a significant dose- and time-dependent upregulation of both DeTyr-tub and Acetyl-tub in MCF10A, MCF7, and MDA-MB-231 cell lines.

### 3.2. Microtentacle Positivity Increases following Hydrogen Peroxide Treatment

**Figure 4:**
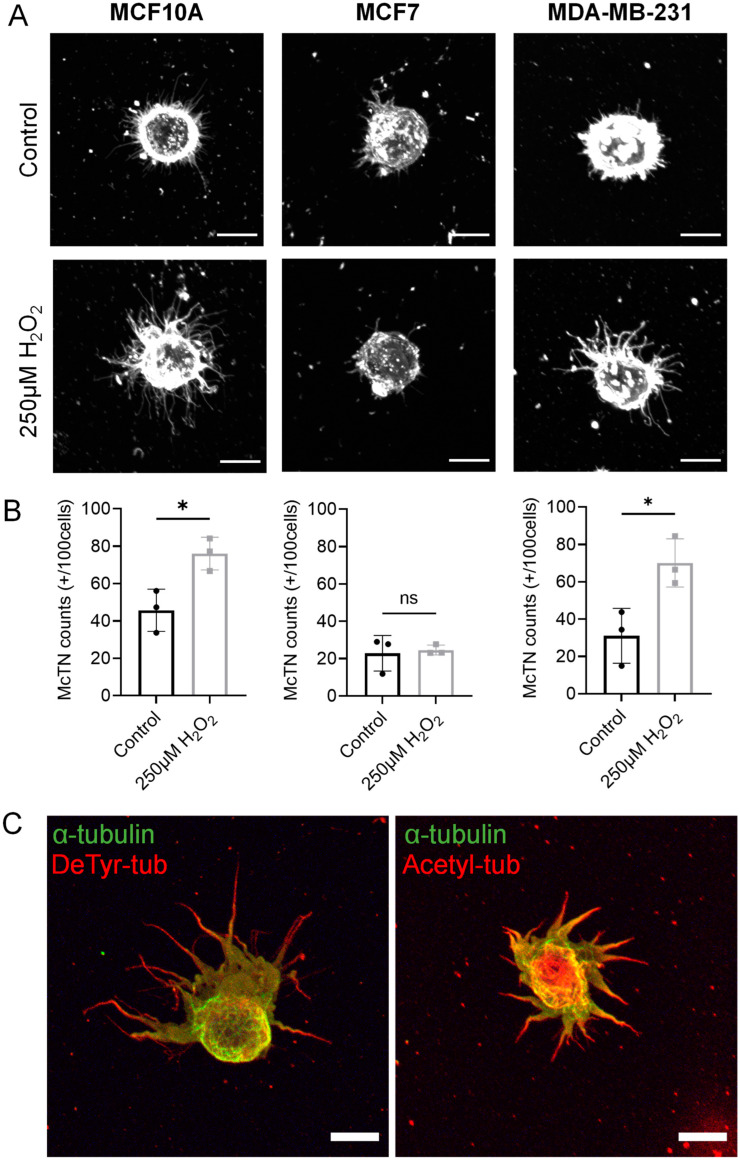
Hydrogen peroxide treatment increases McTN positivity in MCF10A and MDA-MB-231 cells. (**A**) Representative confocal images. MCF10A (left), MCF7 (middle), and MDA-MB-231 cells (right) were treated with control media (top row) or 250 μM H_2_O_2_ (bottom row) for 60 min, tethered, fixed, and stained using fluorophore-conjugated wheat germ agglutinin (WGA, 1:100). Images were captured on an Olympus IX81 microscope with a Fluoview FV1000 confocal laser scanning system at 60× magnification. Scale bar = 10 μm. (**B**) McTN positivity was assessed following control or H_2_O_2_ treatment in MCF10A (left), MCF7 (middle), and MDA-MB-231 cells (right). In three independent experiments, at least 100 cells from each channel, with three technical replicates per condition, were blindly scored and means were compared using an unpaired *t*-test: ns—*p* > 0.05; and *—*p* ≤ 0.05. Data are shown as mean ± SD, n = 3. (**C**) Representative confocal images following immunofluorescence of MCF10A cells treated with 250 μM H_2_O_2_ for 60 min. Left panel: green (α-tubulin); red (DeTyr-tub); and blue (nuclear Hoescht stain). Right panel: green (α-tubulin); red (Acetyl-tub); and blue (nuclear Hoescht stain). Images were captured on an Olympus IX81 microscope with a Fluoview FV1000 confocal laser scanning system at 60× magnification. Scale bar = 10 μm.

Once the appropriate treatment conditions were established, we investigated the functional significance of H_2_O_2_ treatment on cancer phenotypes. Previous studies demonstrated that microtentacles (McTNs) are microtubule-based cellular protrusions composed predominantly of DeTyr-tub and/or Acetyl-tub [19,23]. Since H_2_O_2_ induces both α-tubulin detyrosination and acetylation, we hypothesized that treatment would result in a higher percentage of cells within a population producing McTNs. To test this assumption, adherent MCF10A, MCF7, and MDA-MB-231 cells were treated for 60 min with control media (HBSS) or 250 μM H_2_O_2_. Following treatment washout, cells were detached, counted, and tethered onto TetherChips [30]. TetherChip technology allows for the capture of cells in a free-floating state, permitting fixation and fluorescent labeling of cell membranes and membrane-based McTNs for downstream analysis. Visualization of McTNs using a fluorescent WGA-based membrane stain revealed that H_2_O_2_-treated MCF10A and MDA-MB-231 cells appeared to have more numerous McTNs than control (HBSS)-treated cells (Figure 4A). No visual difference was observed in McTNs between control and H_2_O_2_-treated MCF7 cells (Figure 4A). Blinded, binary scoring of cells as positive or negative for McTNs revealed a statistically significant increase in McTN positivity between H_2_O_2_-treated and control-treated MCF10A and MDA-MB-231 cells (Figure 4B). Interestingly, the MCF7 cell line did not exhibit the same increase in McTN positivity in response to H_2_O_2_ treatment, despite the similar induction of DeTyr-tub and Acetyl-tub (Figure 4B). To confirm that the induced McTNs in MCF10A and MDA-MB-231 cells were composed of DeTyr-tub and Acetyl-tub, we performed immunofluorescence on tethered cells. Confocal imaging confirmed that H_2_O_2_-induced McTNs were composed of DeTyr-tub (left) and Acetyl-tub (right) filaments (Figure 4C and Supplemental Figure S1). These results demonstrate that both non-tumorigenic MCF10A cells and metastatic MDA-MB-231 cells respond to elevated H_2_O_2_ by increasing formation of McTNs composed of DeTyr-tub and Acetyl-tub, while the weakly tumorigenic MCF7 cells do not.

### 3.3. Hydrogen Peroxide Inhibits Reattachment with Limited Effects on Viability

**Figure 5:**
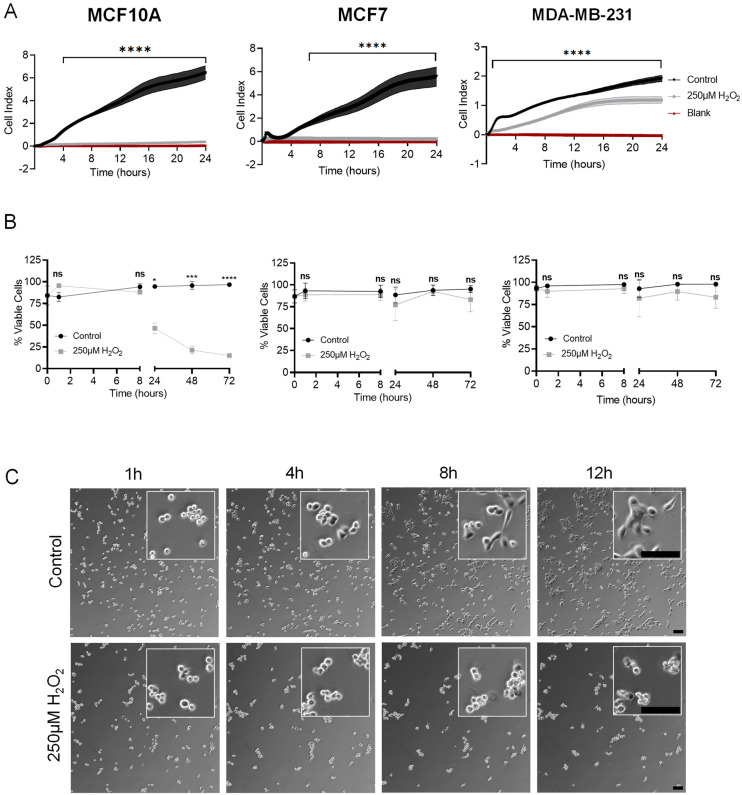
Hydrogen peroxide inhibits reattachment in MCF10A, MCF7, and MDA-MB-231 cells. (**A**) Representative xCelligence assay traces. Real-time impedance measurements were taken every 5 min over 24 h; data are shown as mean ± SD, n = 3. Control (HBSS) and 250 μM H_2_O_2_ were compared using a two-way ANOVA with Bonferroni’s multiple comparisons test: ****—*p* ≤ 0.0001. (**B**) Trypan blue exclusion assay was performed at the indicated time points to assess cell viability before and after treatment, which was washed out at T = 1 h. All samples were measured at least twice; data are shown as mean ± SD, n = 3. Data were compared using a two-way ANOVA with Sidak’s multiple comparisons test: ns—*p* > 0.05; *—*p* ≤ 0.05; ***—*p* ≤ 0.001; and ****—*p* ≤ 0.0001. (**C**) Phase contrast time-lapse images were taken of MCF10A cells at indicated time points following treatment on a Nikon Ti2E microscope at 10× magnification. Scale bar = 100 μm.

Given the significant increase in McTN positivity in two out of the three cell lines tested, we examined the effects of H_2_O_2_ on metastatic phenotypes that are known to be facilitated by McTNs. We assayed the reattachment potential of cells following a one-hour pre-treatment with 250 µM H_2_O_2_. Real-time electrical impedance-based measurements revealed a statistically significant inhibition of cellular reattachment in all three cell lines in the H_2_O_2_-treated cells compared to control (HBSS) cell populations (Figure 5A). Since H_2_O_2_ increases McTNs, this finding was an unexpected result. To confirm that these results were not an artifact of increased cell death, cell viability was assessed via a trypan blue exclusion assay. Measurements taken at the start of the experiment (T = 0), after treatment washout (T = 1 h), and at subsequent time points out to 72 h revealed no significant induction of cell death in H_2_O_2_-treated cells out to 8 h, by which time statistical significance is already reached in the xCelligence recordings (Figure 5B and Supplemental Figure S2). Induction of cell death was observed in the MCF10A cell line 24 h following treatment (Figure 5B). However, there was no significant difference in cell death between control and H_2_O_2_-treated populations in the MCF7 or MDA-MB-231 cell lines, even out to 72 h (Figure 5B). These data suggest non-tumorigenic cells do not tolerate H_2_O_2_ treatment for long periods of time, while the tumorigenic and metastatic cells remain unaffected. Given that the same reattachment effects were observed in all three cell lines despite differences in viability, the data exclude the possibility that cell death is causing the robust reattachment inhibition. To confirm our reattachment results using an orthogonal approach, time-lapse microscopy images were taken to assess cellular reattachment to standard tissue culture-treated dishes. In all three cell lines, control cells began to reattach by 4 h, with the majority of cells appearing to have reattached by 12 h (Figure 5C and Supplemental Figure S3). In contrast, the H_2_O_2_-treated cells did not appear to reattach, with the majority of cells remaining in suspension even at 12 h (Figure 5C and Supplemental Figure S3). These results suggest that H_2_O_2_-induced increases in McTN formation do not directly translate to enhanced function.

### 3.4. Homotypic Cell Cluster Formation Is Decreased through Hydrogen Peroxide Treatment

**Figure 6:**
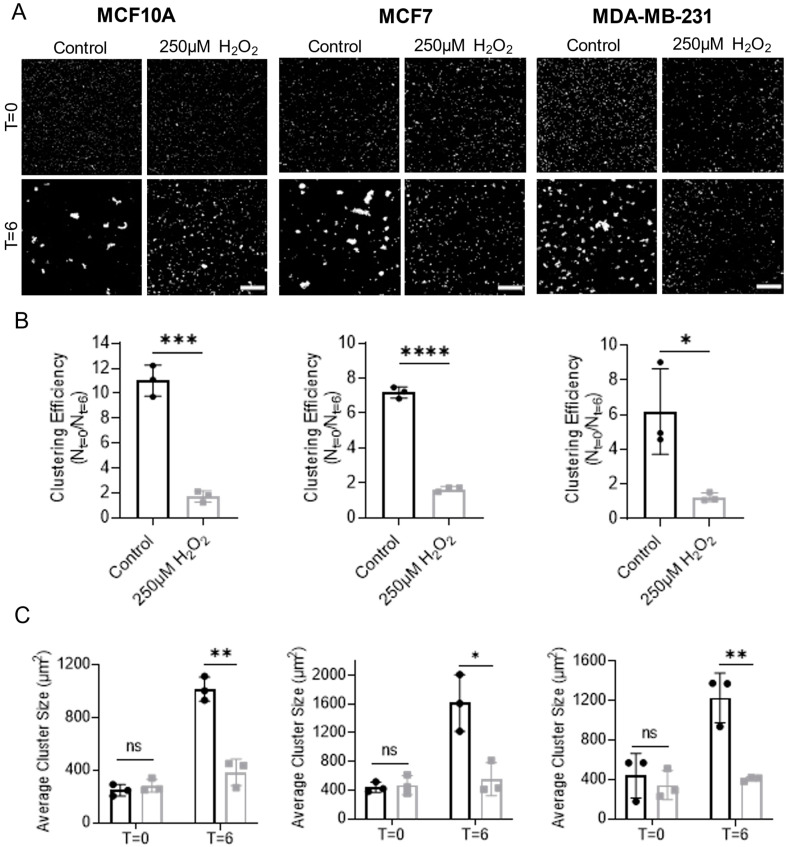
Hydrogen peroxide decreases homotypic cell clustering efficiency in MCF10A, MCF7, and MDA-MB-231 cells. (**A**) Representative images of cells and cell clusters following live cell nuclear staining and loading onto PEM only-coated Ibidi slides at T = 0 (top row) and T = 6 h following treatment (bottom row). Images were acquired on a Nikon Ti2E microscope at 4× magnification. Scale bar = 500 μm. (**B**) Cluster numbers were quantified in ImageJ and clustering efficiency was calculated by dividing number of objects (i.e., cells and/or clusters) at start of suspension (T = 0) by number of clusters after six hours in suspension (T = 6). Data are shown as mean ± SD, n = 3, with three technical replicates per n. Statistical analysis was performed using an unpaired *t*-test: *—*p* ≤ 0.05; ***—*p* ≤ 0.001; and ****—*p* ≤ 0.0001. (**C**) Cluster size was measured in ImageJ at T = 0 and T = 6 for each condition at each time point. Data are shown as mean ± SD, n = 3, with three technical replicates per n. Statistical analysis was performed with multiple unpaired *t*-tests; ns—*p* > 0.05; *—*p* ≤ 0.05; **, *p* ≤ 0.01.

Based on the finding that H_2_O_2_-induced McTNs were not functionally aiding in cellular reattachment, we probed whether H_2_O_2_ treatment would affect another McTN-mediated process: homotypic cell clustering. Our data revealed significantly reduced cluster formation in H_2_O_2_-treated MCF10A, MCF7, and MDA-MB-231 cells compared to control-treated cells. Cluster formation (or lack thereof) was observed visually by utilizing a live cell nuclear stain (Figure 6A). Quantification of clustering efficiency was performed by dividing the number of objects (clusters) at the start of the experiment (T = 0) by the number at the end of the experiment (T = 6) for each condition. As cells form clusters, there will be a smaller number of objects at T = 6 than T = 0, resulting in a higher number for clustering efficiency. Our results showed a statistically significant decrease in clustering efficiency in all three cell lines in H_2_O_2_-treated cells compared to control cells (Figure 6B). As a secondary metric, we also assessed the average cluster size at T = 0 and T = 6. This analysis showed that there was no significant difference in the size of single cells and/or clusters at the start of the experiment between control and H_2_O_2_-treated populations (Figure 6C). However, the average cluster size was significantly lower in the H_2_O_2_-treated populations compared to control across all three cell lines at T = 6, confirming that smaller clusters were formed when cells were pre-treated with H_2_O_2_ (Figure 6C). Cumulatively, these data support a model in which H_2_O_2_ treatment may increase McTN formation but the McTNs are not functionally promoting cellular reattachment or clustering. 

## 4. Discussion

H_2_O_2_ is a well-known second messenger in cells [5,8,31]. By oxidizing cysteine residues, H_2_O_2_ modulates the function of many proteins that regulate cellular growth, metabolism, and viability [5,32]. The normal intracellular concentration of H_2_O_2_ is between 1–100 nM [5], while levels in the TME are much higher, with one report stating that levels may reach 50–100 µM [7]. Effects of elevated H_2_O_2_ include DNA damage, proliferation, angiogenesis, resistance to cell death, and reprogramming of immune and stromal cells in the TME [4,6,10]. However, there is conflicting evidence that elevated H_2_O_2_ promotes cancer cell death [10]. One explanation is that cancer cells gain a selective advantage from elevated H_2_O_2_ up to a certain point, which varies by cell type, but elevation beyond this threshold can be cytotoxic [10]. This study investigated the role of elevated ROS in the TME through specifically probing whether H_2_O_2_ would “push” non-tumorigenic and weakly tumorigenic mammary epithelial cells toward a more metastatic phenotype and examined the effects on an already metastatic cell line: MDA-MB-231 breast cancer cells. Our cell culture system and TetherChip technology [30] permitted stepwise investigation into the roles of ROS on multiple steps of the metastatic cascade, including detachment and McTN formation, cell clustering, and eventual reattachment. Moreover, our approach allowed examination of the short-term phenotypes that, though often overlooked by long-term studies examining primary tumor growth or metastasis, are critical to determining cell fate. 

The effects of H_2_O_2_ on the microtubule network have been understudied in the cancer field. However, several reports have connected ROS, microtubule modifications, and calcium as mediators of mechanotransduction in the context of muscle and bone. This pathway, termed X-ROS, was first described in cardiac muscle cells by Prosser, et al. in *Science* in 2011. This seminal work demonstrated how mechanical stimulation leads to microtubule-dependent activation of the membrane protein NOX2, resulting in ROS production and Ca^2+^ sparks [33]. The X-ROS pathway was subsequently found to operate in skeletal muscle [34] and bone [35] and it was demonstrated that detyrosinated and acetylated microtubules mediate mechanotransduction [36,37]. Recently, this pathway conservation was extended to mammary epithelial cells, with ROS generated via mechano-activation of NOX2 acting on TRPM8 channels to promote Ca^2+^ influx [38]. Our results demonstrate that ROS in the cellular microenvironment can alter X-ROS signaling mechanisms. We report here that H_2_O_2_ induces α-tubulin detyrosination and acetylation, which are two microtubule modifications critical for mediating mechanotransduction [36,37] (Figure 1, Figure 2 and Figure 3). Future studies will be needed in order to define the role of X-ROS calcium signaling in response to our H_2_O_2_ stimulus.

Homeostatic mechanotransduction relies on cells properly sensing and responding to mechanical cues in their environment. A critical change in the mechanical microenvironment that occurs during metastasis is the detachment of cells from the primary tumor [39]. Our group has repeatedly demonstrated that cellular detachment results in the formation of microtubule-based protrusions, termed microtentacles (McTNs) [19,22,23,24,30,40,41]. In the current study, we found that non-tumorigenic MCF10A cells and the metastatic MDA-MB-231 cells both exhibited a significant increase in McTN positivity following H_2_O_2_ treatment and detachment, while the weakly tumorigenic MCF7 cells did not (Figure 4). There are several potential explanations for this finding. For example, upregulation of antioxidant systems is a frequent occurrence in cancer cells and could block ROS-dependent mechanical signaling [8,9]. Additionally, chronically elevated H_2_O_2_ in the TME may desensitize cancer cells to mechanical stimuli. However, we note that the MDA-MB-231 cells responded similarly to the MCF10A cells with increased McTN formation, supporting the notion that ROS effects are highly dependent on the genetic background of the specific cell type. We also note that it is only possible to directly correlate the McTN number with metastatic potential within the same genetic background, as has been previously demonstrated [26,27]. Therefore, future studies would be needed to elucidate the molecular basis for differential McTN response to ROS across a larger array of cell lines. 

Functionally, McTNs have been shown to promote cellular reattachment [23] and cell clustering [24]. This study is the first report of a significant induction of McTNs (Figure 4) in two out of the three lines tested that is accompanied by a significant decrease in cellular reattachment (Figure 5) and clustering (Figure 6) in all three cell lines. To explain this discordance, we hypothesize that McTN formation and function are dependent on a two-step process. The first step is microtubule-based extension of the McTN protrusions. It has been shown that McTNs are formed when microtubule stabilization, through detyrosination and/or acetylation, overcomes suppression by the actin cortex. Specifically, weakening the actin cortex through inhibition of Rho-associated kinase (ROCK) led to increased McTN formation [42], while ML-7-mediated chemical stabilization of the actin cortex reduced McTN formation [43]. Reorganization of septin proteins also perturbs the actin cortex and increases McTN formation [44]. Once McTNs have formed and suspended cells make contact either with another cell or extracellular matrix, we propose that the second step in the two-step model is dynamic contraction of the actin cortex to bring the cell together with its contact. Although this step remains more technically challenging to experimentally demonstrate, our current results support this potential two-step model, in which H_2_O_2_ increases microtubule stabilization and McTN formation but may hinder actin contractility. This model may explain our observed decrease in cellular reattachment and clustering, despite the increase in McTNs.

Production of H_2_O_2_ is therapeutically relevant as it is implicated in the mechanism of action of microtubule-stabilizing taxane compounds, a common first-line therapy for metastatic breast cancer [45,46]. While effective at reducing tumor growth, emerging evidence suggests that taxanes may inadvertently increase metastasis [16,47,48]. Neoadjuvant taxane use has been shown to slow tumor growth but increase circulating tumor cells and disseminated metastases in mice, as well as increase secondary metastasis sites in human patients [16]. In addition to changes in the TME, taxanes have also been reported to alter breast tumor cells themselves, which could increase metastatic potential. Taxanes have been shown to increase DeTyr-tub [15,40] and Acetyl-tub protein expression [49], as well as induce cell death by increasing accumulation of hydrogen peroxide [46]. In line with these data, we report that exposure to H_2_O_2_ leads to significant induction of DeTyr-tub and Acetyl-tub in three independent cell lines. Further elucidation of how microtubule-stabilizing compounds affect ROS production and the impact on metastasis will provide fertile ground for future investigation.

This study has demonstrated that exposing non-tumorigenic, weakly tumorigenic, and metastatic breast cancer cells to elevated H_2_O_2_ induces a significant elevation of α-tubulin detyrosination and acetylation (Figure 1, Figure 2 and Figure 3). While the dose of H_2_O_2_ (250 μM) required to induce significant α-tubulin detyrosination and acetylation was slightly higher than that observed in the in vivo tumor microenvironment (50 μM to 100 μM), we also recognize that local variations across the microenvironment could potentially result in individual cells encountering slightly higher levels. These microtubule modifications are enriched in McTNs, associated with a more invasive phenotype, and are both poor prognostic indicators in breast cancer patients [19,20,21,22]. As expected, we saw significantly increased McTN positivity in the MCF10A and MDA-MB-231 cell lines but not in the MCF7 cell line (Figure 4), raising the possibility that certain cancer cell lines have adaptive mechanisms to counteract oxidative stress. Furthermore, we report a significant decrease in cellular reattachment (Figure 5) and cluster formation (Figure 6), indicating that H_2_O_2_-induced McTNs are not fully functional. These data establish for the first time that microtentacle formation can be separated from the functions to promote reattachment and clustering, which indicates that there are functional steps that remain to be identified. 

## Data Availability

The data generated in this study are available within the article and its Supplementary Data files. Raw data are available from the corresponding author upon request.

## Supplementary Materials

The following supporting information can be downloaded at: https://www.mdpi.com/article/10.3390/cells12091266/s1, Figure S1: H_2_O_2_-induced McTNs in MCF10A and MDA-MB-231 are composed of DeTyr-tub and Acetyl-tub; Figure S2: Independent xCelligence Reattachment Replicates; Figure S3: Time-lapse microscopy videos demonstrate H_2_O_2_ inhibits reattachment in MCF7 and MDA-MB-231 cells; Figure S4: Densitometry bar graphs used to perform statistics for Figure 1A,B; Figure S5: Densitometry bar graphs used to perform statistics for Figure 2A,B. All uncropped immunoblot replicates are included in a separate PDF.
