# Supplementary material for: Hydrogen Peroxide Induces α-Tubulin Detyrosination and Acetylation and Impacts Breast Cancer Metastatic Phenotypes
Source: Cells. 2023 Apr 27;12(9):1266. doi: 10.3390/cells12091266 (PMC10177274; doi:10.3390/cells12091266)
Supplement: Supplementary file 1 [file cells-12-01266-s001.zip › cells-2331737-supplementary.pdf]

Cells 2331737

Supplemental Information

Supplemental Figure S1

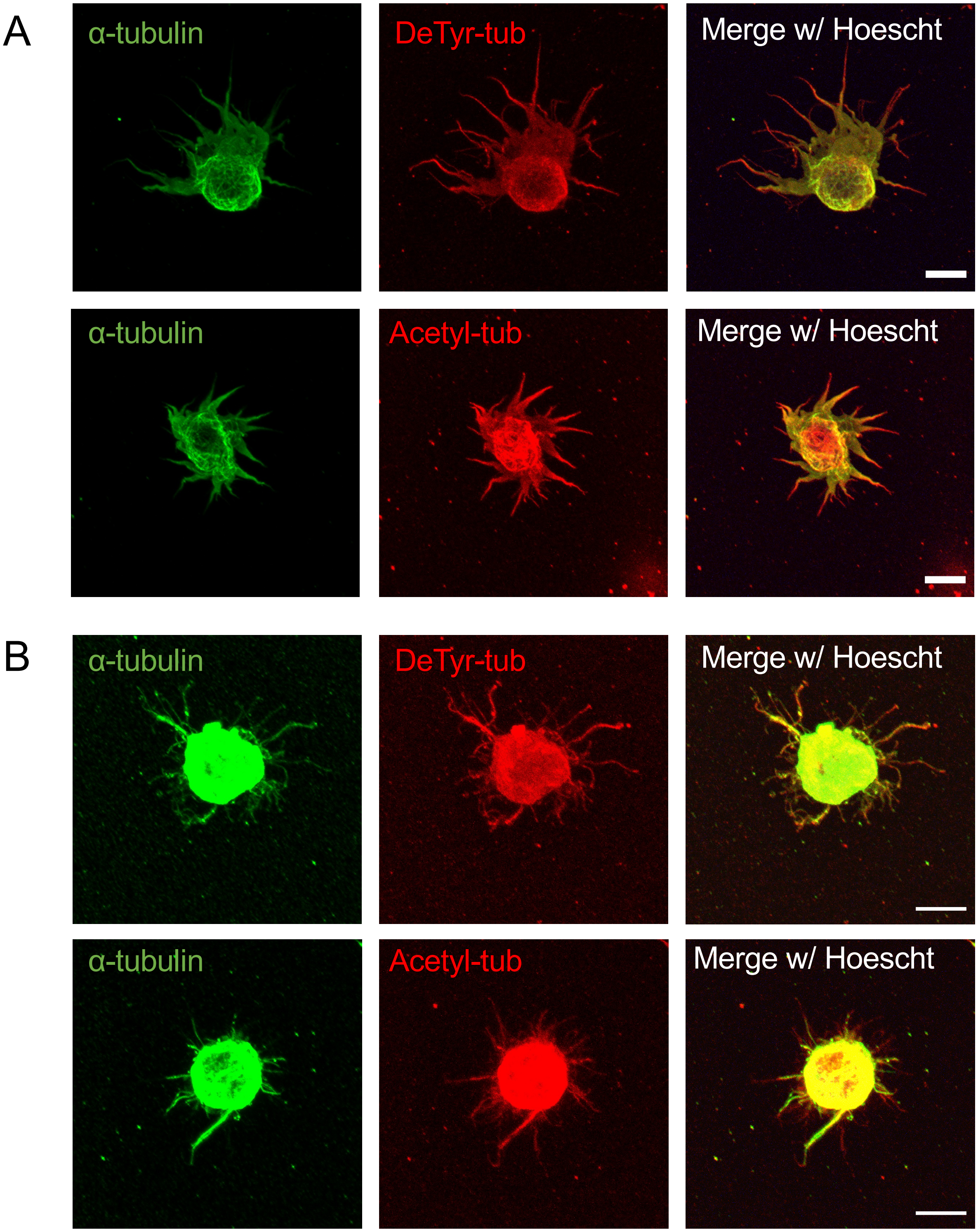

<u>Figure S1</u>: H_2O_2-induced McTNs in MCF10A and MDA-MB-231 are composed of DeTyr-tub and Acetyl-tub.

Representative immunofluorescence images of MCF10A **(A)** and MDA-MB-231 **(B)** cells treated with 250μM H_2O_2 for 60 minutes. For both A and B, top row: Left panel (green = α-tubulin), Middle panel (Red = DeTyr-tub), Right panel (merged with blue nuclear Hoescht stain). Bottom row: Left panel (green = α-tubulin), Middle panel (Red = Acetyl-tub), Right panel (merged with blue nuclear Hoescht stain). Images were captured on an Olympus IX81 microscope with a Fluoview FV1000 confocal laser scanning system at 60X magnification. Scale bar = 10μm.

Supplemental Figure S2

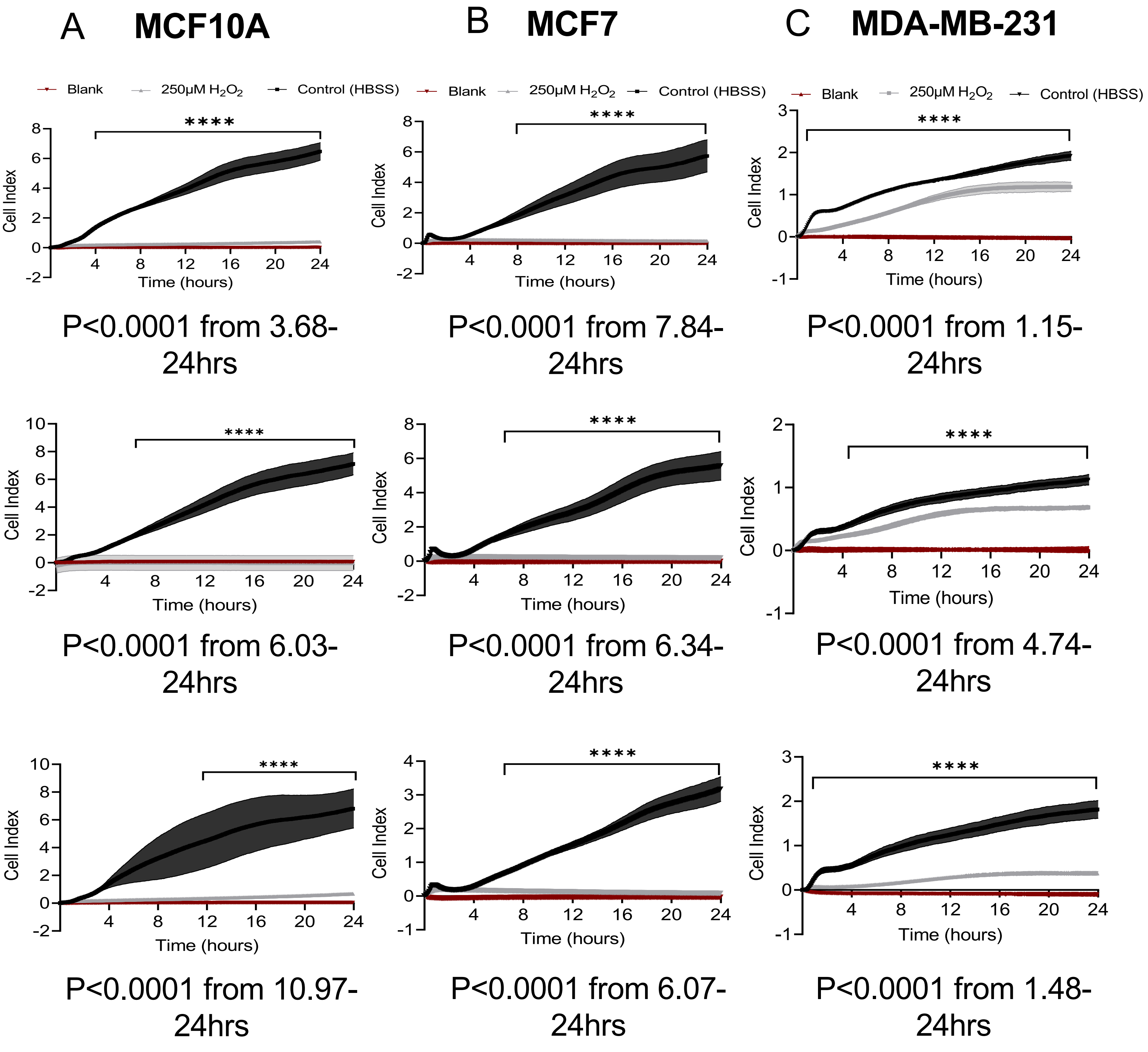

Figure S2: Independent xCelligence Reattachment Replicates.
Three independent xCelligence assay experiments from MCF10A **(A)**, MCF7 **(B)**, and MDA-MB-231 **(C)** cell lines. Real-time, impedance measurements were taken every 5 minutes over 24 hours, data are shown as mean ± SD, n = 3. Red = Blank, Black = Control (HBSS), Grey =250μM H_2O_2. Control and 250μM H_2O_2 were compared using a two-way ANOVA with Bonferroni's multiple comparisons test, ****, $p \leq 0.0001$.

Supplemental Figure S3

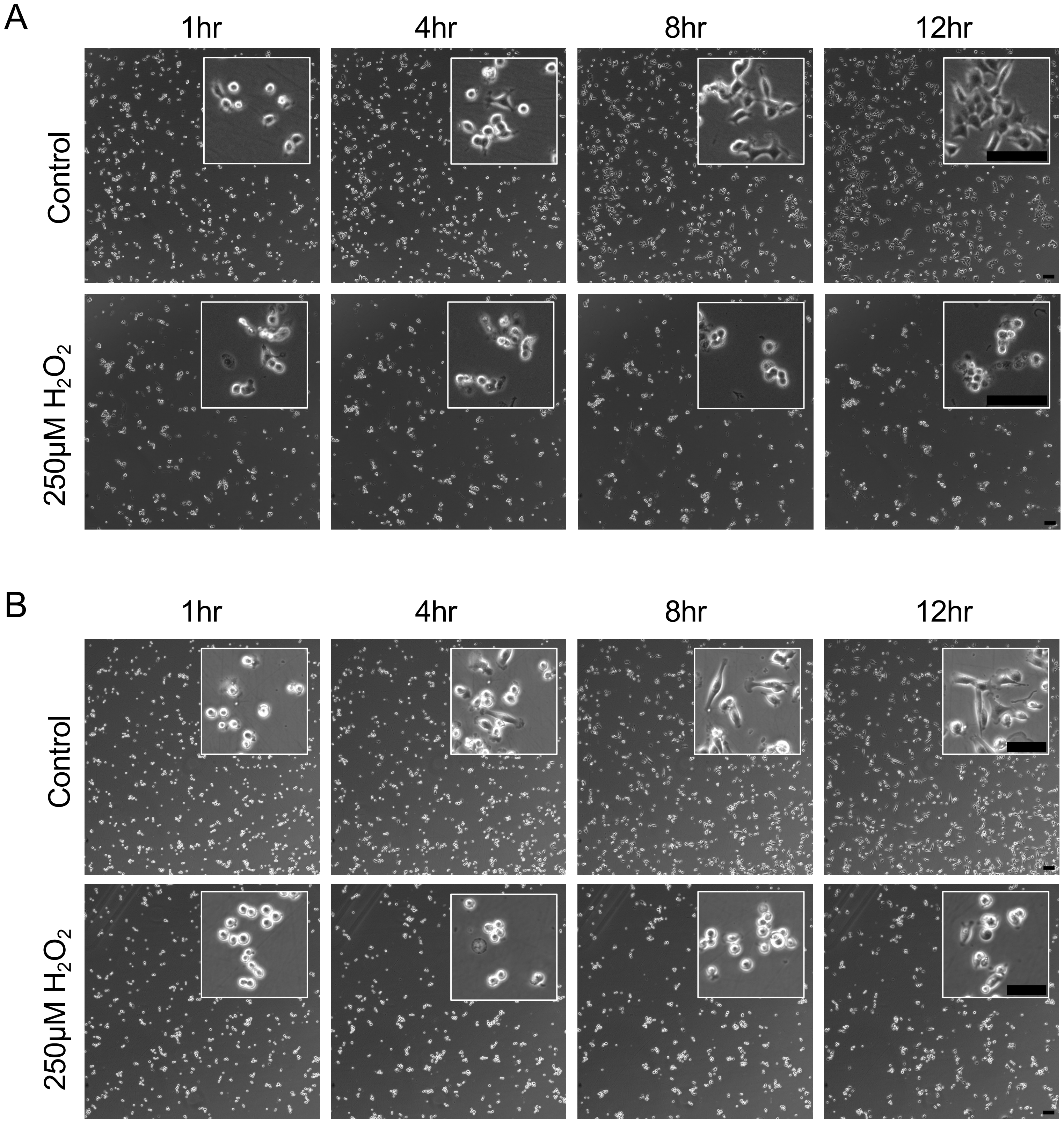

<u>Figure S3</u>: **Time-lapse microscopy videos demonstrate H_2O_2 inhibits reattachment in MCF7 and MDA-MB-231 cells.**

A) Phase contrast time-lapse images were taken of MCF7 cells at indicated time points following treatment on a Nikon Ti2E microscope at 10X magnification. Scale bar = 100μm.

B) Phase contrast time-lapse images were taken of MDA-MB-231 cells at indicated time points following treatment on a Nikon Ti2E microscope at 10X magnification. Scale bar = 100μm.

Supplemental Figure S4

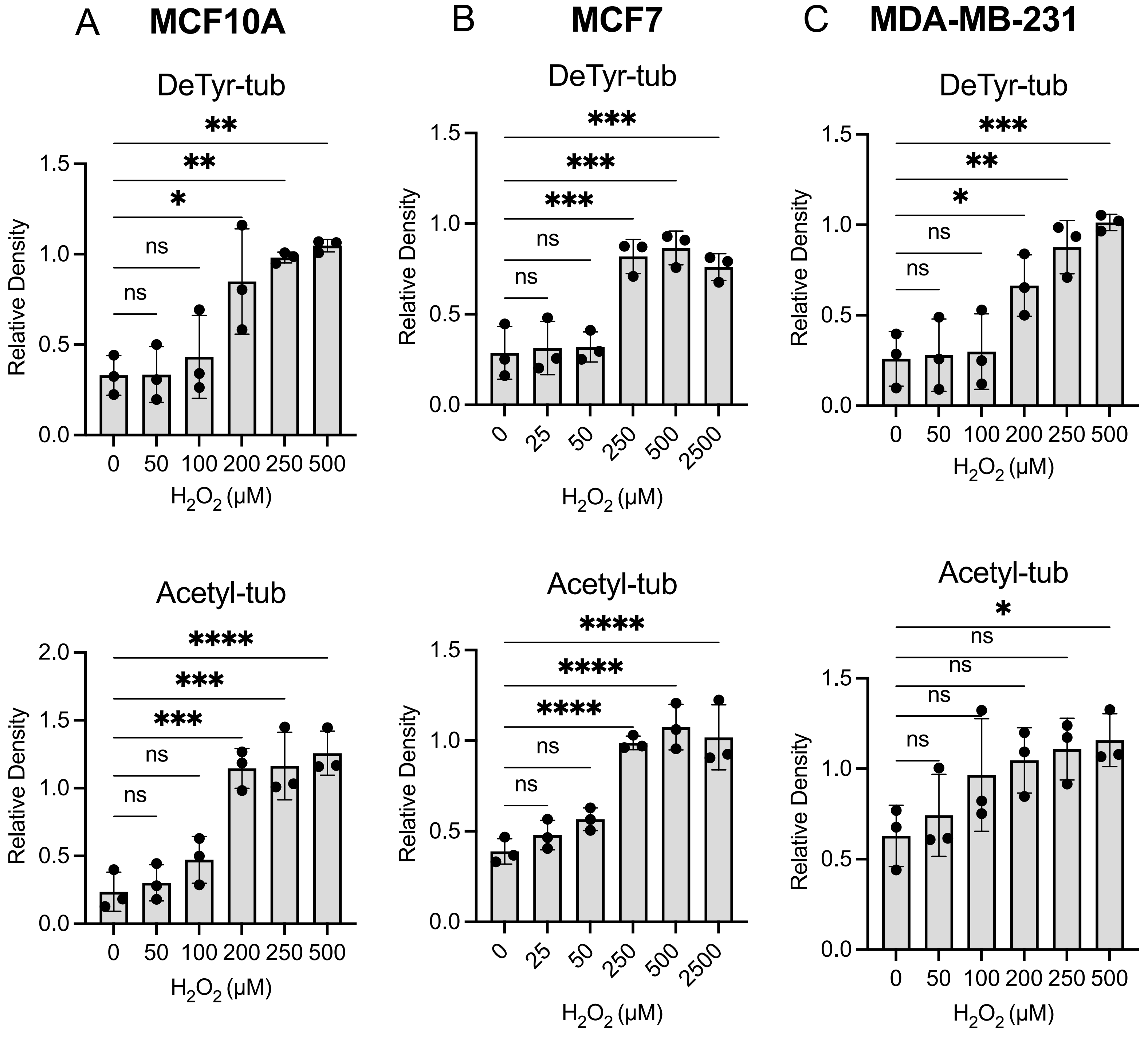

<u>Figure S4</u>: Densitometry bar graphs used to perform statistics for Fig. 1A-B.
Bar graphs used to perform statistical analysis on densitometry corresponding to H_2O_2 dose response immunoblots in Figures 1A-B. Top row shows DeTyr-tub relative density following normalization to total α-tubulin in MCF10A **(A)**, MCF7 **(B)**, and MDA-MB-231 **(C)** cell lines. Bottom row shows Acetyl-tub relative density, following normalization to total α-tubulin, in MCF10A **(A)**, MCF7 **(B)**, and MDA-MB-231 **(C)** cell lines. All means were compared using a one-way ANOVA with Dunnett's multiple comparisons test, ns, non-significant; *, $p \leq 0.05$; **, $p \leq 0.01$; ***, $p \leq 0.001$; ****, $p \leq 0.0001$.

Supplemental Figure S5

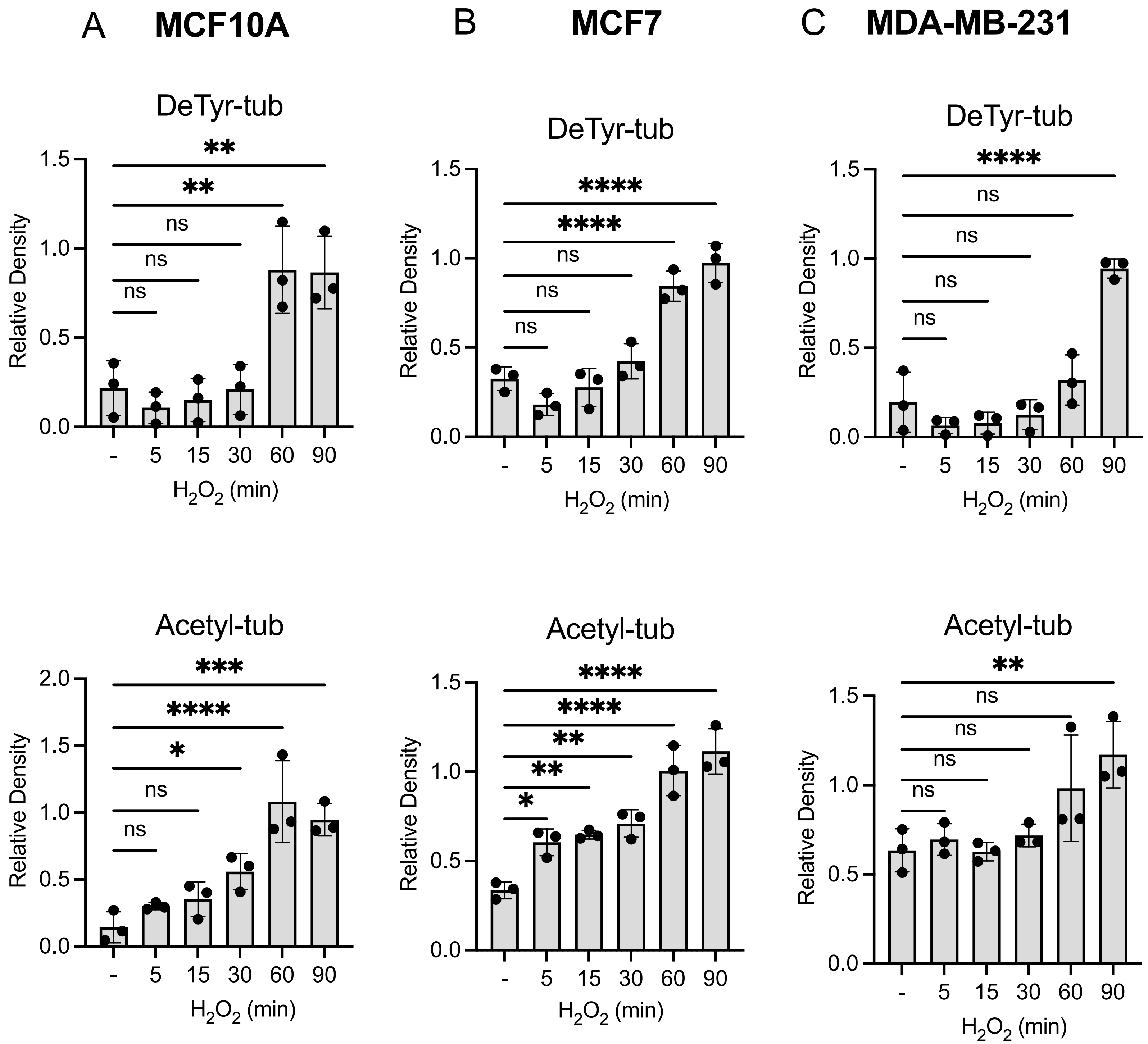

<u>Figure S5</u>: Densitometry bar graphs used to perform statistics for Fig. 2A-B.
Bar graphs used to perform statistical analysis on densitometry corresponding to H_2O_2 time response immunoblots in Figure 2A-B. Top row shows DeTyr-tub relative density, following normalization to total α-tubulin, in MCF10A **(A)**, MCF7 **(B)**, and MDA-MB-231 **(C)** cell lines. Bottom row shows Acetyl-tub relative density, following normalization to total α-tubulin, in MCF10A **(A)**, MCF7 **(B)**, and MDA-MB-231 **(C)** cell lines. All means were compared using a one-way ANOVA with Dunnett's multiple comparisons test, ns, non-significant; *, $p \leq 0.05$; **, $p \leq 0.01$; ***, $p \leq 0.001$; ****, $p \leq 0.0001$.
